# The Effect of Different Parameters on the Development of Compressive Strength of Oil Palm Shell Geopolymer Concrete

**DOI:** 10.1155/2014/898536

**Published:** 2014-10-28

**Authors:** Ramin Hosseini Kupaei, U. Johnson Alengaram, Mohd Zamin Jumaat

**Affiliations:** Department of Civil Engineering, Faculty of Engineering, University of Malaya, 50603 Kuala Lumpur, Malaysia

## Abstract

This paper presents the experimental results of an on-going research project on geopolymer lightweight concrete using two locally available waste materials—low calcium fly ash (FA) and oil palm shell (OPS)—as the binder and lightweight coarse aggregate, respectively. OPS was pretreated with three different alkaline solutions of sodium hydroxide (NaOH), potassium hydroxide, and sodium silicate as well as polyvinyl alcohol (PVA) for 30 days; afterwards, oil palm shell geopolymer lightweight concrete (OPSGPC) was cast by using both pretreated and untreated OPSs. The effect of these solutions on the water absorption of OPS, and the development of compressive strength in different curing conditions of OPSGPC produced by pretreated OPS were investigated; subsequently the influence of NaOH concentration, alkaline solution to FA ratio (A/FA), and different curing regimes on the compressive strength and density of OPSGPC produced by untreated OPS was inspected. The 24-hour water absorption value for OPS pretreated with 20% and 50% PVA solution was about 4% compared to 23% for untreated OPS. OPSGPC produced from OPS treated with 50% PVA solution produced the highest compressive strength of about 30 MPa in ambient cured condition. The pretreatment with alkaline solution did not have a significant positive effect on the water absorption of OPS aggregate and the compressive strength of OPSGPC. The result revealed that a maximum compressive strength of 32 MPa could be obtained at a temperature of 65°C and curing period of 4 days. This investigation also found that an A/FA ratio of 0.45 has the optimum amount of alkaline liquid and it resulted in the highest level of compressive strength.

## 1. Introduction

The worldwide consumption of cement has risen to around 2.6 billion and the use of virgin materials in the production of cement has had negative impact on the environment [1]. Further, large amount of energy is consumed in the production of cement and as a result, cement industry has become one of the largest contributors of carbon dioxide (CO_2_). Research works on minimizing the use of cement through waste materials such as fly ash (FA) and ground granulated blast furnace slag (GGBS) produced positive outcome. Alkaline-activated binders are produced by the activation of different natural materials and industrial by-products such as FA, metakaolin, GGBS, kaolinitic clays, rice husk ash (RHA), and red mud are being used in the development of cementless concrete [2].

Many researchers focussed their attention on the use of FA as an environmental friendly material to replace cement in concrete. FA is a kind of by-product derived from the combustion of pulverized coal and collected by mechanical and electrostatic separators from the fuel gases of power plants [3]. FA is the residue of power plant furnaces and is formed from mineral substances of particles and mainly alumino-silicate-based ceramic spheres with a lesser number of iron-rich spheres [4]. The origin of the coal and how it was combusted are factors that will determine the final properties of coal FA [5]. According to the ASTM C618, FA can be classified as being either Class F or Class C; fly ash with Class C contains higher levels of calcium [6]. Fly ash with lower levels of calcium is preferred for geopolymers because high amount of calcium can impact the process of polymerization and change the microstructure of the final geopolymer [7].

Recently geopolymer concrete brought the attention of many researchers worldwide. The use of industrial by-products such as FA, GGBS, RHA, metakaolin, silica fume, limestone powder, and shale oil ash in the development of geopolymer mortar and the concrete is becoming more common [8]. In order to achieve a suitable chemical composition in the development of geopolymers, the preferred method is to blend FA with another high silica material [9–11]. In previous studies, researchers made use of metakaolinite to obtain geopolymers by reaction with alkaline metal (Na or K) silicate [12–14]. For the calculation of different types of geopolymer composites, it is important that geopolymer skeleton network is formed through different kinds of materials (sand, mica, etc.). This property opens the possibility to appropriate the quality of geopolymer composite to the substitute material [15]. The production of geopolymers takes place by polycondensation and can start from a variety of raw materials. Geopolymeric materials are attractive because of excellent mechanical properties and durability [16]. Furthermore, due to much lower Ca content, geopolymer-based materials are much more resistant to acid attack than Portland cement based ones [17]. The applications of geopolymer-based materials include a wide range of products such as new ceramics, cements, matrices for hazardous waste stabilization, fire-resistant materials, asbestos-free materials, and high-tech materials [18]. Recent literatures stated that geopolymer mortar can be used as a repair material since the bonding between geopolymer mortar and substrate materials was high enough compared to commercial repair materials [19].

Olivia and Nikraz [20] investigated the effects of aggregate content, alkaline solution to FA ratio (A/FA), sodium silicate to sodium hydroxide ratio, and curing method on FA based geopolymer concrete. They reported that geopolymer concrete can be produced with a 28-day compressive strength of 55 MPa. The specimens had higher tensile and flexural strength, produced less expansion and drying shrinkage, and showed 14.9–28.8% lower modulus of elasticity than the OPC control mix. Nazari et al. [21] considered the factors that affect the compressive strength and proposed a suitable procedure for producing OPC geopolymer. In the previous works by Riahi et al. [22, 23], the main factors affecting the compressive strength of ash based geopolymers include the particle size of the utilized ash, curing temperature, curing time, and NaOH concentration. The binder in geopolymer concrete is different from that in OPC concrete; the effect of the interaction between the aggregates and the geopolymer binder was investigated by Sarker et al. [24]. Even though aggregate constitutes major volume in geopolymer concrete, only limited study related to this parameter has been reported.

The use of waste materials by construction industry for sustainable development is gaining worldwide attention and a lot of waste materials are being used or recycled. The use of recycled aggregates in pervious geopolymer concrete (PGC) was studied by Sata et al. [25]. They reported that both the concrete aggregate and crushed clay bricks from the demolished structural concrete member can be used as recycled coarse aggregates for making PGC with acceptable properties. Joseph and Mathew [26] conducted a study on the influence of aggregate content on the engineering properties of geopolymer concrete. Recently, there was an attempt by Kupaei et al. [27] to utilize local industrial waste known as oil palm shell (OPS) as coarse aggregate in oil palm shell geopolymer concrete (OPSGPC). They reported that mix design for the geopolymer concrete produced with OPS differs widely from the procedure used for mix proportioning of concrete using conventional lightweight and normal weight aggregates.

In Malaysia and few other South East Asian countries, the production of palm oil results in a lot of industrial waste products, including OPS. The annual production of OPS is approximately 4.56 million tonnes and these are dumped in the factory yards or used as fuel to operate generators. Many researchers in South Asia and African countries have been researching to replace the conventional crushed granite aggregate with OPS as lightweight aggregate [28–32]. The mix design for OPS concrete (OPSC) has resulted in concrete with sufficient strength, as specified for structural lightweight concrete, and has given satisfactory workability with superplasticizer [30]. Alengaram et al. [29] reported an *E* value in the range of 5.5–11 GPa for the OPSC. Further, the aggregate interlock property of OPS enabled higher shear strength of OPSC than the conventional concrete made with crushed granite aggregate [31]. Alengaram et al. [33] reported that the thermal conductivity of 0.45 W/m °K (Watts/metre Kelvin) for OPSC lies within the range of 0.05 and 0.69 W/m °K of other lightweight aggregate concrete. Shafigh et al. [34] presented a new method using crushed OPS to produce OPSC and reported 28- and 56-day compressive strength of about 53 and 56 MPa, respectively. Though many researchers focussed their attention on the mechanical, functional and structural aspects of OPSC, there is hardly any evidence on the effect of chemicals on OPS; only Mannan et al. [35] reported the effect of different chemicals on the OPS and found that polyvinyl alcohol (PVA) had a positive effect due to an increase in the compressive strength of about 41% compared to OPSC with untreated OPS in the OPSC. Since OPS is being used in geopolymer concrete as coarse aggregate [27], the effect of alkaline activators on OPS is vital.

The mechanical properties of concrete are strongly affected by the bond between cement paste and aggregate at the interfacial transition zone (ITZ) [36]. There are good correlations between microstructure characteristics of ITZ and compressive strength [37]. Brough and Atkinson [36] studied the ITZ in alkali-activated slag cement paste. They reported no enhanced interfacial porosity due to filling up the ITZ by the hydration products in sodium silicate-activated slag cement mortars. Demie et al. [37] conducted an experimental study on the correlations between compressive strength development and ITZ in self-compacting geopolymer concrete. They reported that improved performance of concrete was found when the compressive strength increased through formation of dense ITZ between the aggregate and binder matrix at higher superplasticizers (SP) dosage.

Geopolymers require rather longer heat curing times to develop their strength. A 53% increase in strength has been recorded after the geopolymer was cured using heat [38]. Because water is a fundamental component of alkaline activation reactions, the geopolymer curing process becomes more important [39]. The presence of humidity during the curing process can affect the structural and mechanical properties of alkaline activated FA pastes, mortars, and concretes [40]. Typically, 70% of the compressive strength is developed during the first 12 hours of the curing process [41]. To reach its optimum strength, geopolymer concrete should be cured at temperatures ranging from 40°C to 80°C for at least 6 hours [42, 43], although Škvára et al. [44] have claimed that compressive strength can continue to grow for several years. Temuujin et al. [45] suggested that the curing temperatures used during the first 4 to 48 hours are critical for the manufacture of geopolymers. Palomo et al. [43] found that reactions in FA based geopolymers are accelerated by curing temperatures. Conversely, Hardjito et al. [46] claimed that higher curing temperatures do not guarantee higher compressive strength.

Elevated curing temperatures give geopolymers ceramic like properties and additional benefits [47]. The strength of geopolymers increases after exposure to elevated temperatures [48] and this means that its resistance to fire should be better compared to concrete that uses Portland cement, which loses a substantial amount of its strength after being exposed to temperatures greater than 800°C [38]. Kani et al. [49] examined hydrothermal curing at elevated temperatures; they found that efflorescence was reduced when the components were not rich in aluminum.

This investigation focuses on the effect of different types of alkaline solutions on the OPS. The OPS was soaked in alkaline activators of NaOH, potassium hydroxide (KOH), and sodium silicate of 14 M and the effect of these activators on the water absorption and compressive strength in two curing conditions of oven-cured and ambient cured specimens was investigated. Further, the effect of PVA on the OPS was also investigated and reported. The use of organic aggregate such as OPS in geopolymer concretes makes different characteristics and behavior for them, compared to the use of normal aggregates, and there is no research on the effect of heat curing on the compressive strength of OPSGPC. With these considerations, further objectives of the present study were (i) to obtain the effect of different ratios of alkaline activator solution-to-FA, (ii) to investigate the effect of different molarities of alkaline activators, and (iii) to determine the effect of curing period (1, 1.5, 2, 3, 4, 5, 8, and 10 days) and temperature (room temperature of 30, 50, 65, 80, 95, and 110°C) on the compressive strength of OPSGPC. The OPSGPC was prepared using class F-FA as binder, pretreated and untreated OPS as coarse aggregate, and suitable alkaline activators.

## 2. Materials

### 2.1. Fly Ash

FA was used as the main binder in this investigation and its chemical composition was determined by X-ray fluorescence (XRF) analysis and expressed as a percentage of the overall mass of the constituent oxides as shown in Table 1. The FA used in this study was obtained from Lafarge Malayan Cement Bhd, Malaysia. As shown in Table 1, the FA used in this study had a very low percentage of carbon, as indicated by the low loss on ignition (LOI) values. The Si-to-Al ratio was about 2, and the calcium oxide content was also very low. The iron oxide (Fe_2_O_3_) content was relatively low. As a result, the colour of the FA was darker than ordinary Portland cement (OPC), which contains less iron oxide.

**Figure 1:**
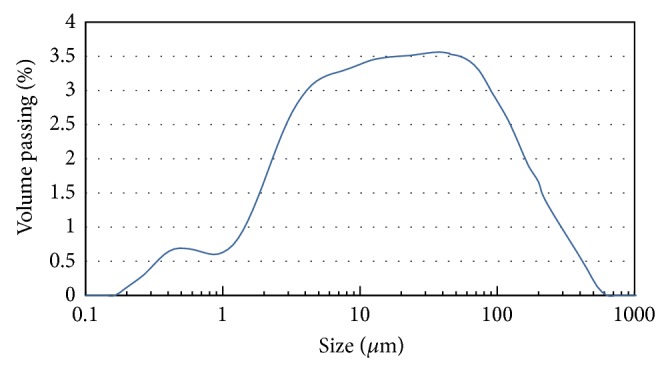
Particle size distribution of fly ash.

**Figure 2:**
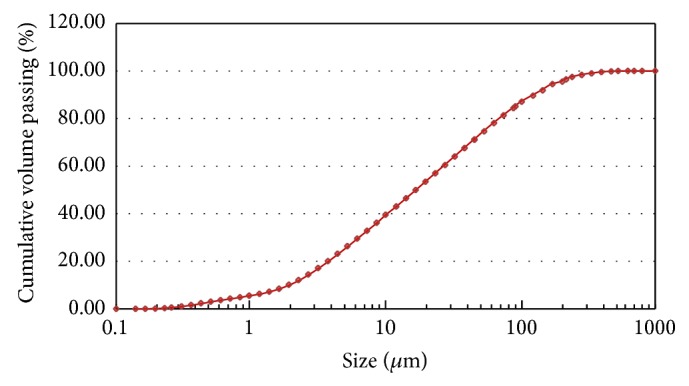
Cumulative particle size distribution of fly ash.

The results of a particle size analysis of the FA revealed that 81.6% of the particles were smaller than 62 *μ*m and fit with the parameters shown in Figure 1. The cumulative particle size distribution of the FA is given in Figure 2.

**Figure 3:**
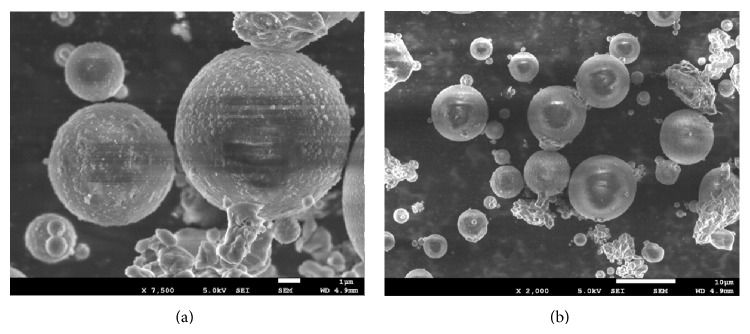
FESEM photography of fly ash particles.

The spherical particle shape results of FESEM analysis of the FA are shown in Figure 3.

### 2.2. Fine Aggregate

The commonly used fine aggregate in Malaysia is mining sand. The 24-hour water absorption of the fine aggregate was found as 0.96% and the only size between 5.0 mm and 300 *μ*m used in this investigation. The specific gravity and fineness modulus of used fine aggregates were 2.68 and 2.73, respectively.

### 2.3. Coarse Aggregate

**Figure 4:**
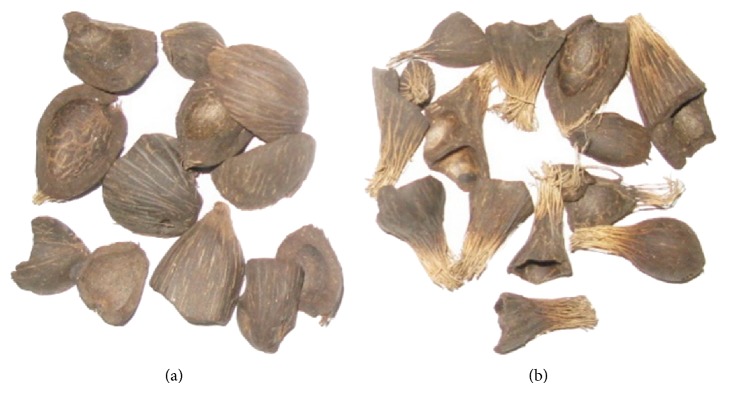
(a) Old OPS without fibres. (b) Fresh OPS with fibres.

**Figure 5:**
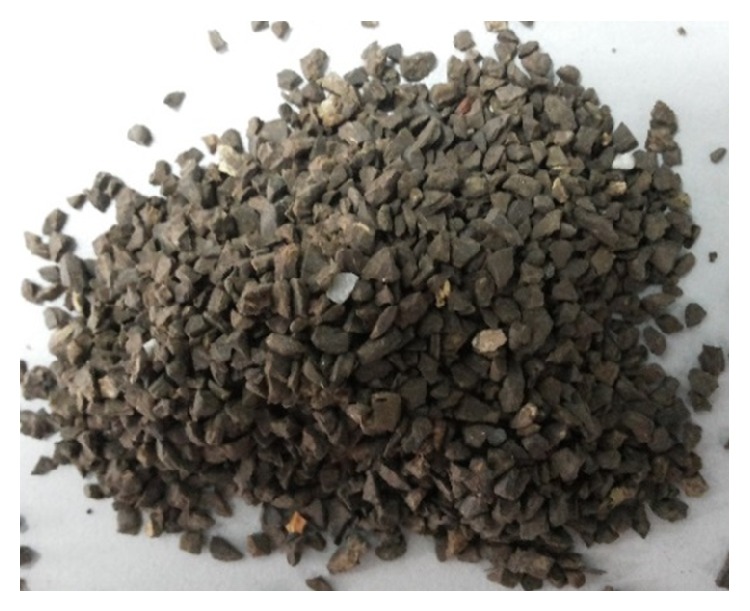
Crushed OPS aggregates with size of 1.18 mm to 5 mm.

The fresh OPS includes fibres as shown in Figure 4(b) which absorb the paste water and decrease the workability and also reduce the contact area between the OPS surface and the mortar, which causes a weak interfacial transition zone [34], so the old disposed OPS in the vicinity of the palm oil factory was used for this investigation. The old OPS obtained from the local palm oil factory had dirt and the size measured up to a maximum of 14 mm. The OPS was washed by detergent to remove dirt, oil, and lump of clay and then dried before crushing into different sizes as shown in Figure 5. The OPS sizes bigger than 1.18 mm were used as coarse aggregate in this investigation. The physical properties and the grading of OPS are shown in Tables 2 and 3, respectively.

### 2.4. Water and Superplasticizer

Potable tap water from the pipeline in the lab was kept in the bucket for 24 hours to release chlorine before it was used. In this study, each mixture contained a naphthalene sulphonated superplasticizer to improve the workability of the concrete mix. Criado et al. [50] discussed the importance of using SP in FA based geopolymer concrete. The total water included the water added to the solution and to the concrete. Therefore, the term water-to-FA (W/FA) ratio includes the total water used in each mixture.

### 2.5. Alkaline Activators

The commonly used activators in geopolymer concretes are a combination of NaOH or KOH and sodium silicate (Na_2_SiO_3_) or potassium silicate. The most effective mixtures contain NaOH and Na_2_SiO_3_ because partially dissolved and polymerized silicon reacts well with other components in the mixture to improve the mortar [51]. Xu and van Deventer [52] claimed that NaOH and Na_2_SiO_3_ improved the process of geopolymerization. In the present study, a combination of NaOH and Na_2_SiO_3_ solution with molarities that ranged from of 8 to 16 M was chosen as the alkaline activator [27].

The NaOH and KOH solution was prepared by dissolving the pellets in water. The mass of NaOH and KOH solids in a solution varies depending on the concentration of the solution expressed in terms of molar, M. For instance, NaOH solution with a concentration of 14 M consists of 14 × 40 = 560 grams of NaOH solids per litre of the solution, where 40 is the molecular weight of NaOH. The mass of NaOH solids was calculated as 431 grams per kg of NaOH solution for 14 M concentration. The similar calculation was done for the KOH solution. KOH solution with a concentration of 14 M consists of 14 × 56.12 = 785.68 grams of KOH solids per litre of the solution, where 56.12 is the molecular weight of KOH. The mass of KOH solids was calculated as 561 grams per kg of KOH solution for 14 M concentration. The composition of the Na_2_SiO_3_ solution used was Na_2_O = 12%, SiO_2_ = 30%, and water 58% by mass. The other characteristics of the Na_2_SiO_3_ solution were specific gravity and viscosity at 20°C of 1.53 g/cc and 400 cP, respectively. The ratio of Na_2_SiO_3_ to NaOH solution was kept constant at 2.5 for all the OPSGPC specimens. The ratio of activator solution-to-fly ash (A/FA), by mass, was changed between 0.2 and 0.55 for different mixes. The total water includes the water added in solution and the water added to concrete. Therefore, the term water to FA (W/FA) ratio implies the total water in the mixes. The combination of sodium silicate and NaOH used as alkaline activator solutions in OPSGPC was kept at room temperature for at least 24 hours before mixing it in the concrete.

## 3. Experiment Programme

Initially, the effect of NaOH, KOH, sodium silicate, and also PVA on the water absorption of OPS, development of compressive strength, and curing condition of OPSGPC produced by pretreated OPS was studied in this investigation. Next, the effect of the molarity of the alkaline activators on the compressive strength of the OPSGPC produced by untreated OPS was investigated. Then, the optimum amounts of alkaline activator-to-FA (A/FA) ratio were determined. Finally, the optimum curing temperature and curing period for the OPSGPC produced by untreated OPS was investigated. The mixture used in this study was 1/0.74/0.66 (FA/Sand/OPS) by weight, with FA content of 480 kg/m^3^, 355.2 kg/m^3^ of sand, and 316.8 kg/m^3^ of OPS. The water and SP contents were 66 kg/m^3^ and 9.5 L/m^3^, respectively, based on our previous work [27].

### 3.1. Pretreatment and Preparation of OPS Aggregates Using Different Chemicals

**Figure 6:**
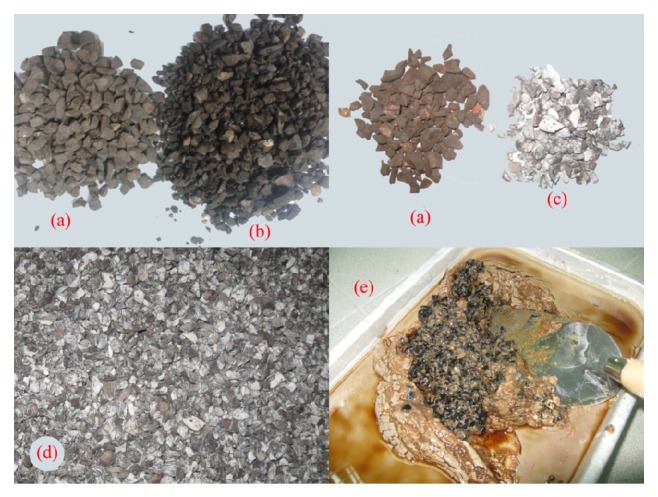
OPS aggregates soaked in (a) water, (b) PVA, (c) NaOH, (d) KOH, and (e) sodium silicate solutions.

The first objective of the work was to study the effect of four different chemicals such as NaOH, KOH, Na_2_SiO_3_, and PVA on OPS. Since OPS used in the OPSGPC has direct contact with the alkaline activators, it is imperative to know the effect of the activators on OPS. PVA solution was prepared by dissolving 100 grams of powder form of PVA in 900 grams of water. It should be noted that liquid form of the Na_2_SiO_3_ solution was used in this investigation. The NaOH solution with a concentration of 14 M was prepared by dissolving 431 grams of NaOH solids into 569 grams of water. Likewise, KOH solution with a concentration of 14 M consists of 561 grams of KOH solids and 439 grams water per kg of the solution was prepared. The OPS was soaked in these chemicals with different percentages for 30 days and then taken out to get dried and to be used in OPSGPC as coarse aggregate. In order to obtain 5%, 20%, and 50% pretreatment solutions per kg, 50, 200, and 500 grams of each of the above mentioned solutions were added to 950, 800, and 500 grams of water, respectively, and used in the pretreatment of OPS.  Figure 6 shows the soaked OPS in four different chemicals. The water absorption of these pretreated OPS was measured and reported.  Table 4 shows the different concentration of the solutions and their designations.

### 3.2. Specimen Preparation for the Effect of Different Pretreatment

The properties investigated in this part of study include the effect of different pretreatment of OPS on the development of compressive strength of OPSGPC produced by pretreated OPS. All the dry materials were mixed in a pan mixer for three minutes and then the alkaline activator solution was added. The wet mixing continued for another four minutes [27]. The concrete was then cast in the 50 mm cube moulds and poured in three phases and each layer was compacted uniformly. For each mix proportion, 24 specimens were cast. Immediately after casting, the specimens along with the moulds were concealed using plastic and then kept in respective curing conditions to prevent evaporation. The average value of three specimens is reported as the compressive strength.

### 3.3. Specimen Preparation for the Effect of Curing Method

The properties explored in this part of investigation were the effect of two different curing methods on the development of compressive strength of OPSGPC produced by pretreated OPS. Two curing methods, namely ambient cured and oven-cured were selected to investigate the development of compressive strength. The oven-cured specimens were cured in an oven at 65°C for 48 hours and were taken out and left in room temperature and humidity of 28 to 31°C and 60%, respectively, till the day of testing. The ambient-cured specimens were kept in the room condition with temperature and humidity as stated above till the day of testing. This procedure was adopted based on the method suggested by Hardjito et al. [53].

### 3.4. Preparation of Specimens for Different Molarities

A total of 5 mixtures with molarities ranging from 8 M to 16 M were created to study how different concentrations of NaOH influenced the compressive strength and density of an OPSGPC produced by untreated OPS. Each OPSGPC mixture had an alkaline activator-to-FA (A/FA) ratio of 0.35, water, an OPS with maximum nominal size of 5 mm, a fine aggregate, and FA. These factors were kept constant. The samples were cured in an oven for 48 hours at 65°C [27]. After the specimens were taken out of the oven, they were left at ambient condition of 28 to 31°C temperature and 60% humidity till test day.

### 3.5. Preparation of Specimens for Different A/AF Ratios

A total of eight mixtures were prepared with different ratios of A/FA (0.2, 0.25, 0.3, 0.35, 0.4, 0.45, 0.5, and 0.55 by mass), to investigate the optimum ratio for use in OPSGPCs produced by untreated OPS. All dry materials were mixed in a pan mixer for four minutes before adding the alkaline activator solution. After the activator was added, the resulting compound was mixed for another five minutes. The concrete was cast in 50 mm cube molds and poured in two layers and compacted with steel rods, as described in ASTM C109. The water-to-FA (W/FA) ratio was fixed at 0.37 for each mixture with FA/sand/OPS ratio of 1/0.74/0.66; the quantity of FA, additional water, and SP were 480 kg/m^3^, 66 kg/m^3^, and 9.5 L/m^3^, respectively. The molarity of the alkaline activator was 14 M. For each mixture, 6 specimens were cast. Water evaporation was prevented after casting by immediately sealing top of the molds with a thin, plastic layer. The specimens were cured in an oven for 48 hours at 65°C. After the specimens were removed from the oven, they were left at ambient conditions until test day. The room temperature and humidity were 28 to 31°C and 60%, respectively. The samples were tested after 14 and 28 days, in accordance with ASTM C109. An average from three specimens was used to determine the compressive strength.

### 3.6. Preparation of Specimens for Different Curing Regime

An optimum A/FA ratio of 0.45 was selected for the OPSGPC produced by untreated OPS to investigate the effect of different curing temperatures (room temperature of 28°C, 50, 65, 80, 95, and 110°C) and different periods. All specimens left at the ambient conditions after they were removed from the oven until the test day. They were taken out of the oven after 1, 1.5, 2, 3, 4, 5, 8, and 10 days and cured at room temperature of 28 to 31°C until the test day.

## 4. Results and Discussions

### 4.1. Effect of Different Pretreatment on Water Absorption

**Figure 7:**
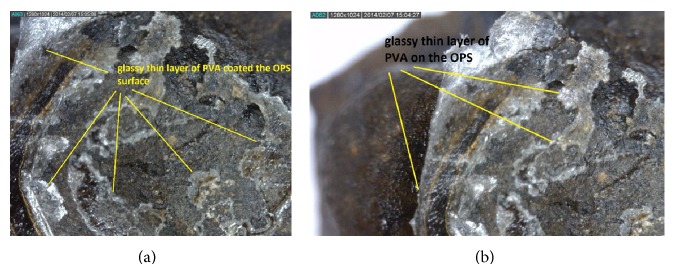
OPS aggregates soaked in polyvinyl alcohol.

Table 4 shows the various types of chemicals and concentration that were used in the investigation of their effect on the OPS. The clear NaOH solution that was used for soaking the OPS turned brown after 3 days. Similar treatment for OPS with KOH solution showed that the solution turned dark brown after 3 days. The surfaces of the OPS aggregates were covered with white colour deposit when subjected to soaking in NaOH and KOH solutions after 30 days. The OPS treated with PVA shows a thin glassy film formed around the OPS as shown in Figure 7. The same as NaOH and KOH alkaline solutions, the OPS aggregates soaked in sodium silicate solution showed similar results. The clear sodium silicate solution became light brown after the aggregates were soaked for 3 days, whereas the solution turned gelatine dark brown form after 5 days of soaking as shown in Figure 6(e). Soaking in alkaline solutions for 30 days has not showed much deterioration on the surface of the OPS aggregates. Mannan et al. also reported that MgSO_4_ solution has some deterioration effect on the OPS aggregates [35]. The water absorption values of both the pretreated and untreated OPS aggregates with different chemicals are shown in Table 5.

The water absorption values of the OPS pretreated with 5%, 20%, and 50% PVA solution were found as 50.04%, 18.07%, and 17.85%, of the untreated OPS, respectively; this could be attributed to the thin film coating by PVA formed around the aggregates that prevent water absorption. The water absorption of the OPS pretreated with 20% and 50% was almost identical and hence it can be inferred that the pretreatment with 20% of PVA solution could be effective.

From Table 5, it can be seen that the effect of PVA is more evident than the alkaline solutions on the reduction of water absorption. The pretreated OPS aggregate with 20% and 50% of PVA solution decreases the water absorption by about 82% of the untreated OPS, while the pretreated alkaline solution of NaOH, KOH, and sodium silicate with the same concentration causes a decrease of about 14%, 17%, and 11%, respectively. Further, it can be seen from Table 5 that the reduction in the water absorption for the pretreated OPS aggregates with NaOH falls between the values of OPS aggregates pretreated with sodium silicate and KOH solutions. However, the pretreatment with KOH has better performance compared to the other two alkaline solutions.

### 4.2. Effect of Pretreatment of OPS on the Compressive Strength

The results of 3-, 7-, 14- and 28-day compressive strength test performed on specimens cured under both oven and ambient curing conditions are reported in Table 6. The cube compressive strengths of ambient-cured specimens at the age of 14 days show that OPSGPC achieves the requisite compressive strength for structural grade lightweight concrete (SLWC) as per ACI 301-10 specifications.

**Figure 8:**
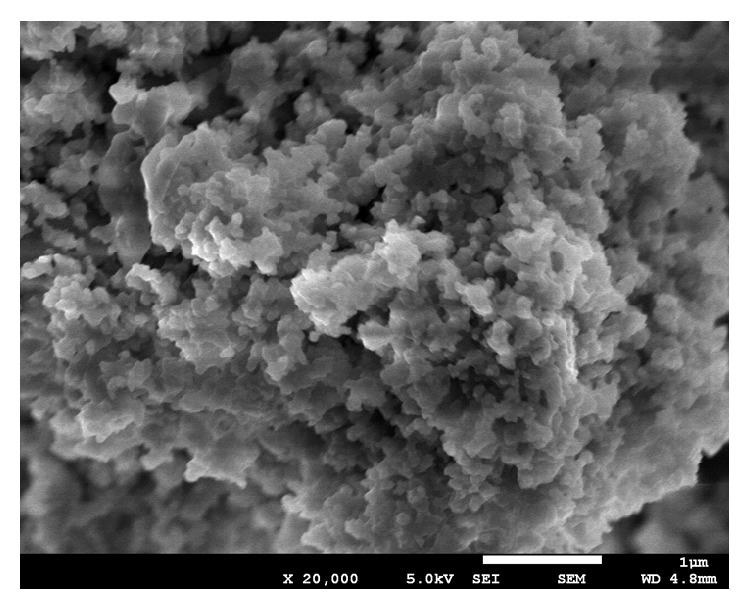
FESEM photography of ambient-cured OPSGPC incorporating pretreated OPS with PVA.

**Figure 9:**
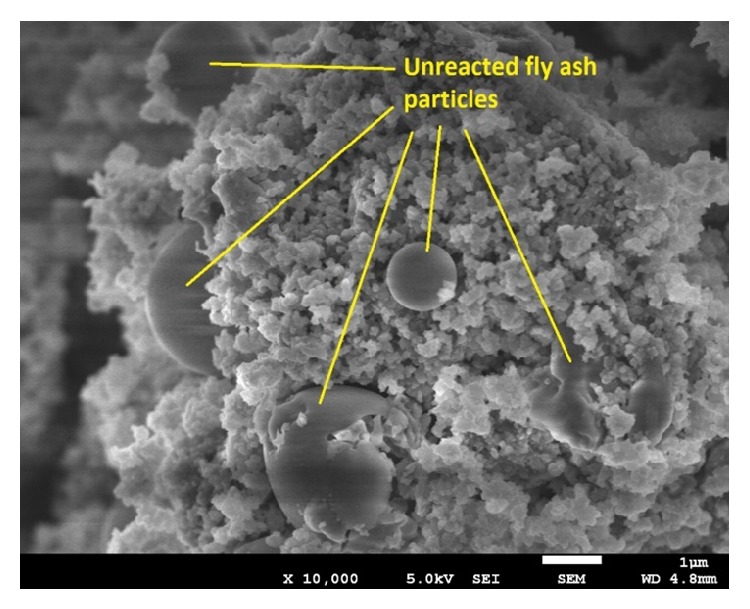
FESEM of ambient-cured OPSGPC incorporating pretreated OPS with KOH.

However, the oven-cured specimens show better performance compared to ambient-cured specimens. The requisite cube compressive strength for SLWC of 20 MPa could be achieved in 3 days for the oven-cured specimens and the 28-day strength was about 32 MPa. The OPSGPC made from the OPS treated with 5% sodium silicate solution showed the lowest compressive strength in ambient-cured condition and that one which made from the OPS treated with 50% PVA solution established the highest compressive strength; however, the compressive strength of both specimens with OPS treated with 20% and 50% PVA produced very close results. The difference in the 28-day compressive strength between the OPSGPC specimens produced from the OPS treated with alkaline solutions and the control specimen (Designation E), both cured in ambient condition, was only about 2.7%; however, it was about 12% for the specimens made with OPS treated in 20% and 50% PVA solutions. This might be attributed to the reduction in the water absorption by the OPS as it was used as the aggregate in concrete. Figures 8 and 9 show the FESEM analysis image of ambient-cured OPSGPC including pretreated OPS with PVA and KOH, respectively.  Figure 9 shows unreacted FA particles in geopolymer skeleton which cause a weaker geopolymer structure leading to lower compressive strength. This might be ascribed to the lack of alkaline activator solution due to absorption of OPS aggregates during the geopolymerization process.

In the oven-cured specimens, there was not much difference between the compressive strength at various ages among the OPSGPC specimens pretreated with different percentage of PVA solution. The geopolymerization is a process that takes place in a short duration and the oven curing condition enables this to be achieved [41] compared to the ambient curing condition. This implies that the longer duration of geopolymerization process has a direct effect on the absorption by OPS from the mortar; the longer processing time might cause the OPS to absorb more alkaline activator solution and water and that will have direct effect on the reduction of the concentration of activator in the mortar, thus resulting in reduced compressive strength. It was also observed that the failure of the OPSGPC specimen in compression was not due to the bond failure between the OPS and the mortar, but rather due to crushing of OPS. In the OPS based concrete, the porous nature of aggregate enhanced the bond between OPS and matrix as the fine particles of binder penetrate the pores in the OPS that enhances the bond and hence improves the compressive strength [29]. They reported that improved performance of concrete when the compressive strength increased through formation of dense ITZ between the aggregate and binder matrix at higher superplasticizers (SP) dosage. However, the failure of the specimen is governed by the both the failure of OPS and the bond between the OPS and the matrix. The compressive strength is also governed by the convex and concave surfaces of OPS and the observation on the convex surface leads to the conclusion that its poor binding with matrix reduces the compressive strength. A comparison between the 28-day compressive strength results in Table 6 which depicts that the OPSGPC incorporating OPS pretreated with PVA solution produced the highest compressive strength compared to other pretreatments in ambient-cured condition, while in the case of oven-cured specimens, the highest strength of about 33 MPa was achieved for the OPSGPC produced with the OPS pretreated in KOH solution.

**Figure 10:**
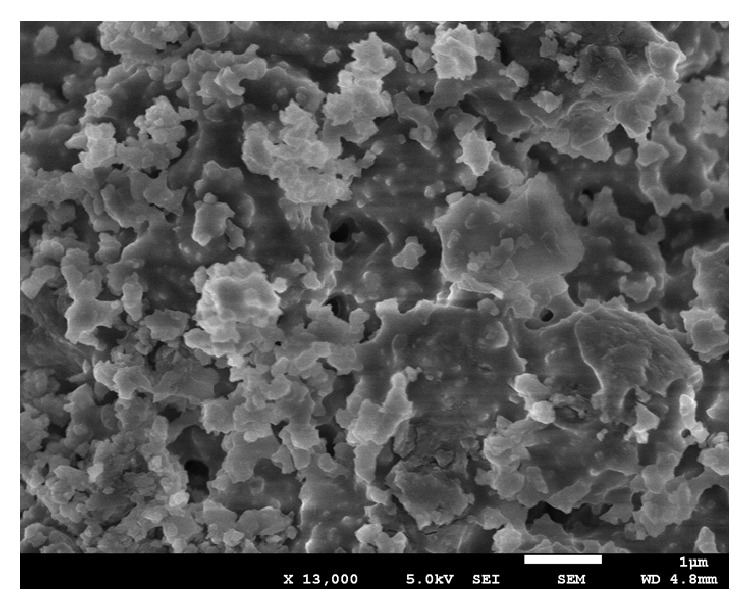
FESEM of ambient-cured OPSGPC incorporating pretreated OPS with KOH.

Figure 10 shows the FESEM analysis image of oven-cured OPSGPC including pretreated OPS in KOH. More condensed geopolymer paste with fewer pores is obvious in this figure which leads to higher compressive strength than the ambient-cured OPSGPCs. This might be attributed to the better geopolymerization process due to oven curing condition. It should be noted that the other specimens pretreated with alkaline and PVA solutions produced comparable strength. It means that pretreatment with alkaline solution in oven-cured condition leads to higher compressive strength in OPSGPC and pretreatment with PVA solution in ambient-cured condition causes higher compressive strength in OPSGPC.

### 4.3. Development of Compressive Strength

The 3-, 7-, and 14-day compressive strengths of ambient-cured OPSGPC specimens prepared with alkaline pretreated OPS were about 36%, 55%, and 73% of 28-day compressive strength, respectively, while for the specimens cured in oven, the corresponding percentages were about 65%, 82%, and 94%, respectively.

The 3-, 7-, and 14-day compressive strengths of ambient cured OPSGPC specimens prepared with PVA pretreated OPS were about 43%, 60%, and 73% of 28-day strength, respectively, whereas for oven cured specimens the corresponding values were about 66%, 80%, and 91%, respectively. For the ambient-cured OPSGPC specimens prepared with treated and untreated OPS, about 73% of 28-day compressive strength was achieved in 14 days and the corresponding value for specimens cured in oven was 94%. It can be concluded that the pretreatment of OPS has not much effect on the development of compressive strength in both ambient and oven cured conditions, compared to the specimens made with untreated OPS within the period of 28 days.

A comparison between the 28-day compressive strength of ambient-cured specimen prepared with alkaline pretreated and untreated OPS shows that OPS pretreated with 5% sodium silicate has negligible effect on the compressive strength; the maximum increase in the compressive strength of about 5.5% was found for the specimen prepared with OPS pretreated with 50% NaOH. Similarly, for the oven cured specimens, the effect of the pretreatment was found negligible with the specimen pretreated with 50% of KOH showing an increase of 5%. Moreover, the comparison between the ambient cured specimens prepared with PVA treated OPS and untreated OPS shows that the 3-, 7-, 14-, and 28-day compressive strengths of the former were about 41%, 22%, 14%, and 12%, respectively, of the strength of the later. Thus, it can be concluded that the PVA treatment has some effect on the compressive strength if the specimens were cured in ambient condition.

### 4.4. Effect of Curing Method on the Compressive Strength

The observation on the ambient-cured OPSGPC specimens prepared based on different types of pretreated OPS showed that the specimens had not hardened early until 12 hours. Nevertheless, the physical observation revealed that the OPSGPC loses its plasticity within the few first hours of preparation of the specimen. However, for the specimens that were covered with the plastic wrapping and cured in an oven at 65°C, the hardening took place in less than 2 hours.

The differences in the compressive strength between the 14- and 28-day for the OPSGPC made with and without treated OPS were 38% and 6.62% in the ambient and oven-cured conditions, respectively. The average differences between the 14 and 28-day strength of the oven-cured specimens for the OPS pre-treated with alkaline solutions and PVA solution were about 6.57% and 9.8%, respectively. To achieve comparable strength to geopolymer concrete, it is necessary to cure geopolymer concrete with elevated temperature curing between 40 and 80°C for at least 6 h [43]. In most cases, 70% of the final compressive strength is developed in the first 12 h [41]. Therefore, most of the geopolymerisation takes place within the first 12 hours of heat curing and hence the compressive strength enhancement of OPSGPC between 14 and 28 days in oven-cured condition was insignificant. A longer curing time improves the polymerisation process resulting in higher compressive strength. The previous study indicated that a longer curing time does not produce weaker concrete [10]. However, the increase in the compressive strength after 48 hours of oven curing is not significant [53].

**Figure 11:**
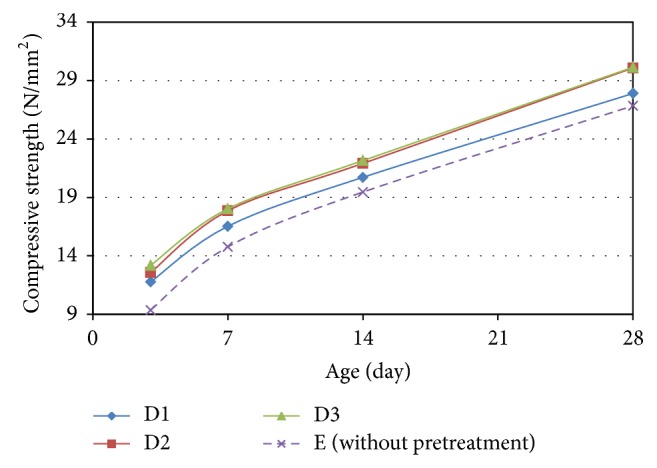
Compressive strength distribution for the OPSGPC with PVA solution treated OPS in ambient-cured condition.

**Figure 12:**
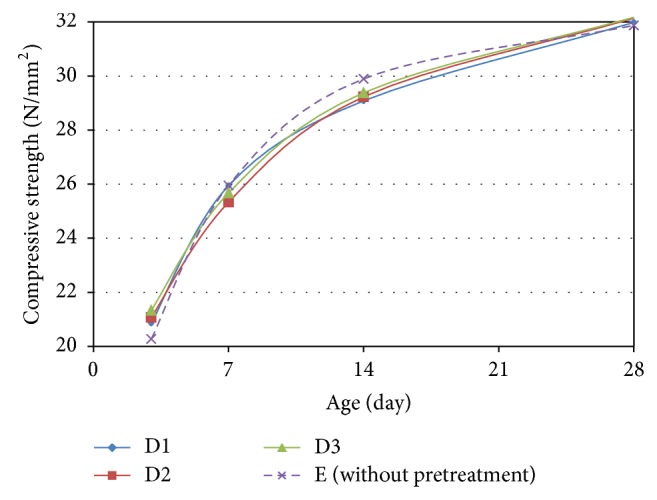
Development of compressive strength for the OPSGPC with PVA solution treated OPS in oven-cured condition.

Figures 11 and 12 show the development of the compressive strength up to a period of 28 days for the OPSGPC prepared with PVA pretreated OPS that was cured in ambient and oven conditions, respectively.

The average increase on the 28-day compressive strength was found to be about 1.25% and 2.72% for the oven and ambient-cured specimens, respectively, compared to the control specimens (designation E). Hence, it can be concluded that the pretreatment of OPS with alkaline solutions had no significant effect on the compressive strength in both the ambient and oven curing conditions; on the contrary, the PVA pretreatment of OPS has some effect on the compressive strength as 12% increase in the average 28-day compressive strength was found for ambient-cured specimens. However, for oven-cured specimens the effect is negligible.

### 4.5. Influence of NaOH Concentration on the Compressive Strength and Density of OPSGPC

**Figure 13:**
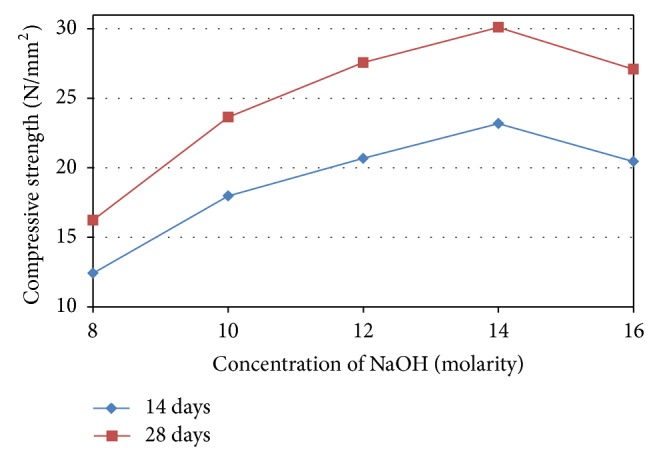
Influence of different concentrations of NaOH on the compressive strength of OPSGPC.

The compressive strength of geopolymers is directly connected to the degree of polymerization, which is strongly influenced by the soluble silicate and aluminate of the geopolymeric system. The dissolution of FA affected by concentration of alkaline solution and could be evaluated by measuring the leaching of Al^3+^ and Si^4+^ ions. Therefore, an important factor in controlling and evaluating the leaching of alumina and silica from FA particles is alkaline concentration, subsequent in geopolymerization and mechanical properties of hardened geopolymer [54]. In general, a higher degree of polymerization in the geopolymeric structures leads to higher compressive strength. The NaOH concentration in the aqueous phase of the geopolymeric process acts on the dissolution process [55]. The use of high concentrations of NaOH leads to greater dissolution of the initial solid materials and increases the geopolymerization reaction resulting in greater compressive strength [56]. Rattanasak and Chindaprasirt [54] conducted a study on the leaching of FA mixed with NaOH by measuring of Si^4+^ and Al^3+^ ions. They reported that Si^4+^ ion concentration for low and high molarity of NaOH was much less than that for the moderate concentration of NaOH. For the high concentration of NaOH, an increase in congealing of silica principally reduced the dissolution.  Figure 13 shows the effect of sodium hydroxide concentrations on the compressive strength of OPSGPC. The mean compressive strength of the three test cubes for each molarity of alkaline activator at the age of 14 days and 28 days are presented in Figure 13. The test results shown in Figure 13 demonstrate that the compressive strength of OPSGPC increased steadily as the concentration of NaOH rose from 8 M to 14 M. A maximum strength of about 30 MPa was obtained at 14 M concentration of NaOH. It declined slightly to 27 MPa when molarity rose to 16 M. Joshi and Kadu reported similar results [57]. The results of their study indicated that a substantial increase in the compressive strength when molarity was varied between 12 M and 14 M.

In general, it was observed that high NaOH concentration in alkaline activator increases the compressive strength of the OPSGPC specimens. Conversely, the compressive strength decreased and it was observed that a part of activator solution remains unreacted and leached out and deposited onto the surface of specimens when the concentration of sodium hydroxide increased to 16 M. In geopolymer process, the concentration of NaOH in the alkaline activator has a significant effect on both the compressive strength and the microstructure of the geopolymers [58]. When an aluminum atom is bonded to four oxygen atoms, a negative charge is created. It is important that a neutral electrical state is sustained for hydroxysodalite geopolymer matrixes in the presence of cations such as Na^+^. Hydroxysodalite mixtures are composed of SiO_4_ and AlO_4_ tetrahedra that are linked together in an alternating fashion by sharing oxygen atoms. In order to counteract the negatively charged aluminum in the tetrahedron hydroxysodalite mixture, positive ions, such as those supplied by Na^+^, K^+^, Li^+^, Ca^2+^, Ba^2+^, NH^4+^, and H_3_O^+^, must be present. These components can be found in the cavities of the framework [59]. A weak dissolution was observed which was consistent with other geopolymerization processes that occurred at low alkaline activators [60]. Hence, an increase in alkaline concentration improved the geopolymerization process, which leads to more compressive strength for the OPSGPC.

As noted, the presence of cations, such as Na^+^, K^+^, and Ca^2+^, influences the state of the electrical charge and the catalytic properties in geopolymer systems [61]. When the NaOH concentration was increased, so too were the Na ions in the system. Subsequently, the Na ions were used to balance the charges and formed alumino-silicate networks [62]. However, the higher alkaline content in the geopolymers promoted greater solid dissolution but excess hydroxide ion concentration caused aluminosilicate gel precipitation in the early stages, hindering further geopolymerization and decreasing strength [63]. The decrease in the compressive strength at 16 M could be related to higher alkaline content in the OPSGPC.

Alonso and Palomo [64] conducted a study on the rate of polymer formation, influenced by parameters such as curing temperature, alkaline concentration, and initial solids content. Their study indicated that high activator concentrations increased the pH in the liquid phase. Consequently, anionic forms of silicate were favoured and polymerization was delayed. They also reported that the resulting pH level was not conducive to stable molecular forms and it was more difficult to form the coagulated structure. They also discovered that NaOH concentrations above 10 M caused lower rates of polymer formation resulting in decreasing mechanical strength.

The oven dry density depends on the sodium hydroxide concentration; higher concentration shows increase in the density. The specimens with a low density of 1744 kg/m^3^ were prepared using 8 M concentration of NaOH but the specimens made with 16 M concentration of NaOH exhibited a maximum density of 1824 kg/m^3^.  Table 7 shows that the oven dry density of the OPSGPC varies from 1744 to 1824 kg/m^3^. The increase in the density for specimens with high concentration of NaOH is attributed to the increase in the viscosity of the solution. In this study, the OPSGPC mixtures with higher levels of sodium hydroxide were more cohesive. The increase in the density of the specimens with 8 to 12 M concentration of NaOH was negligible.

### 4.6. Effect of Different A/FA Ratios on the Compressive Strength of OPSGPC

The compressive strengths of OPSGPC with different A/FA ratios are given in Table 8. For molarities less than 14 M, the rate of geopolymerisation increased as the ratio of A/FA increased from 0.2 to 0.35, increasing the strength of the geopolymer specimens significantly. In contrast, there was only a slight increase in strength when the ratio rose from 0.35 to 0.45. The maximum strength was reached when the A/FA ratio reached 0.45. The results show that the compressive strength of OPSGPC made from a 14 M concentration of NaOH in alkali activator increased as the A/FA ratio increased from 0.2 to 0.45 and reach a peak of about 30 MPa at A/FA ratio of 0.45.

The increase in the compressive strength depended on the nature of the complex chemical geopolymerisation process. The most important factor in the process is the amount of reactive silica because it is a major component of the structural framework of the reaction product that is the result of FA being activated by the alkali activator. Conditions that are highly alkaline dissolve reactive silicates to create polymeric Si–O–Al bonds [65].

One possible explanation for the increase in the compressive strength can be found in the higher amounts of sodium silicate solution. Higher amounts of this solution create more SiO_2_ species, which increases the ratio of SiO_2_/Al_2_O_3_ and more Si–O–Si bonds are formed. Si–O–Si bonds are stronger than Si–O–Al bonds [66] and their presence explains why the strength of geopolymers increases in these situations.

**Figure 14:**
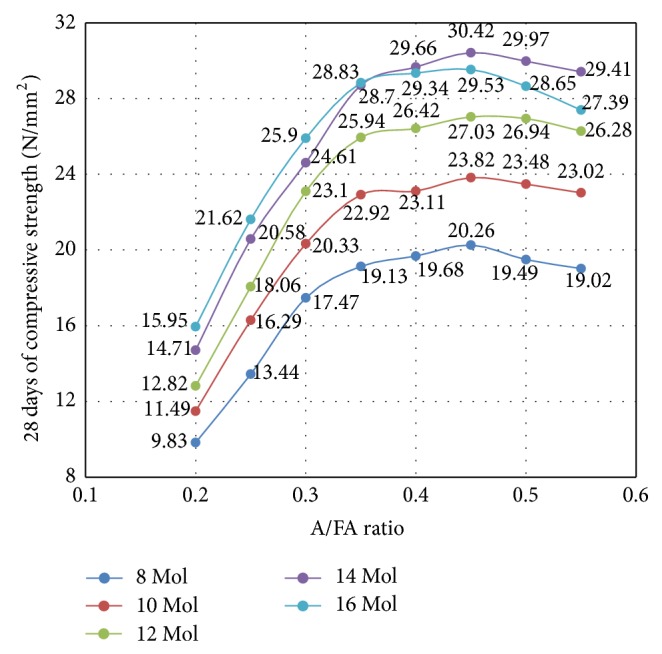
Influence of different A/FA ratios on the compressive strength of OPSGPC at different molarities.

Figure 14 shows the influence of the A/FA ratio on the compressive strength of OPSGPC for different concentrations of NaOH in alkali activator solution. In general, a slight decrease in the compressive strength was noticed for specimens with A/FA ratio from 0.45 to 0.55 as shown in Figure 14.

A possible explanation for the decrease in the compressive strength of OPSGPC may be found in the relationship between the increase in total water content in geopolymer paste and A/FA ratios higher than 0.45. Water is essential in geopolymerisation process, especially for the destruction of solid particles and the hydrolysis of dissolved ions (Al and Si). Water is the reactant in the dissolving part of process, and if the OH^−^ concentration is high enough, then the addition of more water will increase dissolution and hydrolysis. Water also acts as a product in the geopolymerisation process; however, too much water can hinder the geopolymerisation process. Water is a critical factor; too much or too little will affect the geopolymerisation rate, resulting in decreased strength.

### 4.7. Effect of Different Curing Regimes on the Compressive Strength of OPSGPC

**Figure 15:**
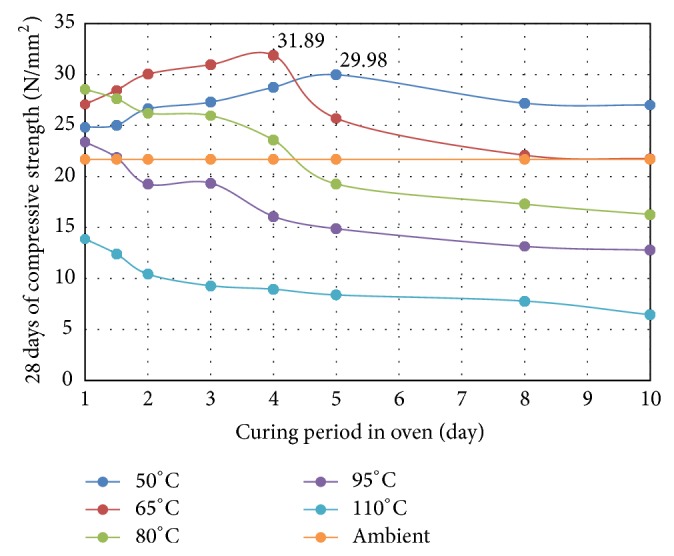
Compressive strength of OPSGPC at different curing temperatures for different curing periods (the specimens were kept in ambient condition after oven curing period till the test day of the 28 days).

The temperature, curing period, and relative humidity are the curing conditions that impact the creation of microstructures and they can affect the mechanical characteristics of alkaline-activated FA [51]. The compressive strength of OPSGPC specimens exposed to different curing temperatures is shown in Figure 15. The specimens cured at 65°C had a maximum compressive strength of about 33 MPa after only 4 days of curing period. In contrast, the lowest compressive strength of 6.45 MPa was obtained for specimen cured at 110°C for 10 days. For specimens cured at 65°C, an increase in curing period up to 4 days causes a steady increase in the compressive strength. However, further increase in curing period beyond 4 days produces less compressive strength. The curing period more than 8 days resulted in a short decrease of compressive strength. However, the compressive strength of specimens gradually decreased when cured at temperatures higher than 65°C.

Hardjito and Rangan [67] found that the compressive strength of FA based geopolymers did not develop significantly when cured at temperatures in excess of 60°C and they recommended that FA based geopolymers should be cured at 65°C. Chindaprasirt et al. [68] conducted a study that looked at the relationship between moisture and the strength produced by the geopolymerization process. They found that the increase in the curing temperature caused their specimens to lose a large amount of moisture, which could undermine the strength of the specimen because the process requires moisture to improve the strength of the final product. Al Bakri et al. [69] used FTIR spectra analysis and found that the higher the Si content of the specimens cured at 60°C, the higher their compressive strength.

Figure 15 also shows the steady raise in the strength for specimens cured at 50°C. Here the peak of about 30 MPa in compressive strength was reached after 5 days of curing period. There was also a slight increase after 1.5 days, followed by a decrease after 5 days of curing period in the compressive strength. However, the decrease of the 10 days of oven curing period was found to be about 10%.

A study was conducted by Rovnaník [70] to study the compressive strength of geopolymers cured at temperatures ranging from 10°C to 80°C. He reported that geopolymers cured at 10°C, 20°C, and 40°C were stronger than those cured at temperatures of 60°C or higher. Figure 5 shows the curing temperatures of 80°C and 95°C resulting in the compressive strength of OPSGPC declining steadily for the first 2 days. The decrease in the compressive strength of specimens cured at a temperature of 110°C for 1 day to 10 days was found to be about 54%.

The results illustrated in Figure 15 indicate that a longer hotter curing period has a negative effect on the strength of the geopolymers, especially for specimens cured at temperatures greater than 65°C. These results are supported by claims made by other researchers [67–69]. For instance, van Jaarsveld et al. [10] claimed that longer and hotter curing times would lead to weaker geopolymer structures. They believed that curing temperatures in excess of 100°C had a negative effect on the geopolymer structures. One explanation for the loss of strength is that the aggregates and the geopolymer matrix expand when the temperature increases [71]. The compressive strength of specimens heat-cured for less than 4 days at temperatures below 80°C was higher than those cured at ambient temperatures.

## 5. Conclusions

The research findings of OPS pretreated with four different chemicals in three different percentages (5%, 20%, and 50%) are presented in this paper. These chemicals were used to investigate the effect of alkaline activators solutions and PVA solution on the water absorption and the compressive strength up to a period of 28 days in two different curing environments. This paper also reports the results of an experimental investigation on the effect of different concentrations of NaOH in the alkaline activator solution, alkaline activator to FA content (A/FA) ratio, and the curing regime on the compressive strength and density of OPSGPC. The molarity of NaOH was varied between 8 M and 16 M; the A/FA ratio and the curing temperature were varied in the range of 0.2 to 0.55 and 55°C to 110°C, respectively. The specimens were cured at different temperatures for the period of 1 day to 10 days and then kept in ambient condition of 28 to 31°C temperature and 60% humidity. Based on the tests and the results obtained, the following conclusions were drawn.

1. The 24-hour water absorption value for OPS pretreated with 20% and 50% PVA was about 4% compared to 23% for untreated OPS. Thus it can be concluded that PVA solution with 20% concentration is sufficient to reduce the water absorption of OPS.
2. The OPSGPC specimens produced using OPS treated with 20% PVA solution produced the 28-day compressive strength of about 30 MPa in ambient-cured condition.
3. Pretreatment with alkaline solution in oven cured condition leads to higher compressive strength in OPSGPC and pretreatment with PVA solution in ambient-cured condition cause higher compressive strength in OPSGPC.
4. The alkaline pretreatment of OPS did not have any significant effect on the compressive strength of OPSGPC in both the ambient and oven curing conditions.
5. The pretreatment of OPS with 20% and 50% PVA solution enhanced the compressive strength of OPSGPC in ambient curing condition by 12% as the PVA coating reduces the water absorption; however, in the oven-cured condition the effect of PVA coating is negligible which could be attributed to early development of geopolymerization.
6. Almost all the specimens achieved about 60% and 80% of the 28-day compressive strengths in 7 days for ambient and the oven-cured specimens, respectively.
7. The effect of concentration of NaOH in alkaline activator (molarity) on the compressive strength showed an increase up to 14 M; however, there is a decrease at 16 M in the compressive strength of OPSGPC.
8. The oven dry density of OPSGPC was found to increase by increasing in the NaOH concentrations.
9. The highest compressive strength of 32 MPa was obtained for the A/FA ratio of 0.45 and higher water content in the A/FA ratio beyond 0.45 reduced the compressive strength.
10. In general, the A/FA ratio between 0.35 and 0.45 is recommended to produce structural grade OPSGPC.
11. Even though the highest compressive strength of 32 MPa was obtained for specimens cured at 65°C and 4 days of curing period, the curing period of 2 days at temperature of 65°C was found to produce compressive strength of 30 Mpa and is recommended for OPSGPC.

## Figures and Tables

**Table 1:** Chemical composition of used fly ash and ordinary Portland cement (%).

Content	SiO_2_	Al_2_O_3_	Fe_2_O_3_	CaO	MgO	SO_3_	K_2_O	Na_2_O	MnO	TiO_2_	P_2_O_5_	Cr_2_O_3_	Sr	Zn	LOI
FA	57.6	28.9	5.79	0.18	0.91	0.23	0.86	0.38	0.035	1.843	0.463	0.016	0.101	0.022	3.63
OPC	19.03	4.6	3.05	63.02	2.64	2.58	1.03	0.26	0.127	0.250	0.030	0.003	0.069	0.009	2.58

**Table 2:** Physical properties of OPS aggregate.

Physical property	OPS
Specific gravity (saturated surface dry)	1.18
Bulk density (compacted) (kg/m^3^)	596
Fineness modulus	5.53
Water absorption (24 h) (%)	22.78

**Table 3:** Grading of OPS aggregates.

Sieve size (mm)	Percentage retained (%)
4.75	10.2
3.54	33.5
2.36	37.2
1.18	19.1

Total	100

**Table 4:** Type of chemicals used for pretreatment of OPS aggregates.

SI	Pretreatment methods	Mass proportion in kg of solution (gr)			Designation
		Solid	Liquid	Water	
1	Pretreated in 5% of 14 M sodium hydroxide solution	21.6	—	978.4	A1
2	Pretreated in 20% of 14 M sodium hydroxide solution	86.2	—	913.8	A2
3	Pretreated in 50% of 14 M sodium hydroxide solution	215.5	—	784.5	A3

4	Pretreated in 5% of 14 M potassium hydroxide solution	28.1	—	971.9	B1
5	Pretreated in 20% of 14 M potassium hydroxide solution	112.2	—	887.8	B2
6	Pretreated in 50% of 14 M potassium hydroxide solution	280.5	—	719.5	B3

7	Pretreated in 5% of sodium silicate solution	—	50.0	950.0	C1
8	Pretreated in 20% of sodium silicate solution	—	200.0	800.0	C2
9	Pretreated in 50% of sodium silicate solution	—	500.0	500.0	C3

10	Pretreated in 5% of PVA solution	5.0	—	995.0	D1
11	Pretreated in 20% of PVA solution	20.0	—	980.0	D2
12	Pretreated in 50% of PVA solution	50.0	—	950.0	D3

13	Without any pretreatment	—	—	—	E

**Table 5:** Water absorption of pretreated and untreated OPS aggregates.

SI	Designation	Water absorption (%)
1	A1	20.14
2	A2	19.85
3	A3	19.35

4	B1	19.88
5	B2	19.43
6	B3	18.76

7	C1	21.29
8	C2	20.91
9	C3	20.04

10	D1	11.30
11	D2	4.08
12	D3	4.03

13	E	22.58

**Table 6:** Compressive strength of OPSGPC in different ages with different pretreated OPS.

Serial number	Designation	Compressive strength (N/mm^2^)							
		Ambient-cured specimens				Oven-cured specimens			
		3-day	7-day	14-day	28-day	3-day	7-day	14-day	28-day
1	A1	9.68	14.91	20.20	27.81	20.86	26.04	30.03	31.90
2	A2	9.89	14.97	20.32	27.84	20.99	26.45	30.25	32.44
3	A3	10.71	15.72	20.84	28.32	21.01	26.67	30.46	32.52

4	B1	9.42	14.96	19.68	27.16	20.85	26.18	30.14	31.92
5	B2	9.88	15.12	20.09	27.86	21.03	26.66	30.33	32.48
6	B3	10.07	15.39	20.14	28.03	21.24	26.89	30.48	32.69

7	C1	9.54	14.95	19.68	26.98	20.68	26.03	30.10	31.89
8	C2	9.82	15.18	19.89	27.02	20.93	26.23	30.24	32.23
9	C3	10.08	15.23	20.01	27.12	21.12	26.39	30.51	32.37

10	D1	11.76	16.53	20.72	27.90	20.85	25.54	29.08	31.98
11	D2	12.56	17.86	21.19	30.08	21.07	25.63	29.23	32.14
12	D3	13.18	18.03	22.15	30.14	21.33	25.77	29.38	32.19

13	E	9.36	14.77	19.46	19.46	20.27	25.95	29.89	31.87

**Table 7:** The density of OPSGPC in different molarities of alkali activator.

NaOH molarities	Density (Kg/m^3^)
8 M	1744
10 M	1753
12 M	1774
14 M	1798
16 M	1824

**Table 8:** Compressive strength of OPSGPC in different A/FA ratios (OPS used in saturated surface dry (SSD) conditions, mix proportion 1/0.74/0.66 (FA/sand/OPS) by weight, FA = 480 Kg/m^3^, W/FA = 0.37, and SP = 9.5 L/m^3^).

Mix order	A/FA ratio	Compressive strength (N/mm^2^)									
		8 Mol solution		10 Mol solution		12 Mol solution		14 Mol solution		16 Mol solution	
		14-day	28-day	14-day	28-day	14-day	28-day	14-day	28-day	14-day	28-day
A1	0.20	6.91	13.04	7.84	14.18	9.15	16.07	13.75	19.84	17.31	23.44
A2	0.25	8.02	15.51	9.98	17.64	11.41	19.26	16.27	23.76	19.49	26.15
A3	0.30	9.46	17.47	11.08	20.33	14.66	23.10	19.09	27.98	20.56	28.58
A4	0.35	9.84	19.13	12.44	22.92	15.97	25.94	21.77	31.12	22.82	30.17
A5	0.40	10.63	19.68	12.84	23.11	16.22	26.42	22.43	31.93	22.91	30.43
A6	0.45	10.84	20.26	13.04	23.82	16.37	27.03	22.57	32.42	21.06	29.53
A7	0.50	10.23	19.49	13.48	23.98	16.91	27.47	21.14	31.08	20.31	28.65
A8	0.55	10.18	19.02	12.49	23.02	16.36	26.94	20.87	30.41	19.29	27.39
